# Evaluation of Serum IL-39 Levels in Patients with Polycystic Ovary Syndrome

**DOI:** 10.3390/jcm15103958

**Published:** 2026-05-20

**Authors:** Mehmet Kagitci, Ilkay Bahceci, Mehmet Kivrak, Sally Al Deseet, Senol Senturk

**Affiliations:** 1 Department of Obstetrics and Gynecology, Faculty of Medicine, Recep Tayyip Erdogan University, 53100 Rize, Turkey; mehmet.kagitci@erdogan.edu.tr (M.K.); hala.al2iam@gmail.com (S.A.D.); senol.senturk@erdogan.edu.tr (S.S.); 2 Department of Medical and Clinical Microbiology, Faculty of Medicine, Recep Tayyip Erdogan University, 53100 Rize, Turkey; ilkay.bahceci@erdogan.edu.tr; 3 Department of Biostatistics and Medical Informatics, Faculty of Medicine, Recep Tayyip Erdogan University, 53100 Rize, Turkey

**Keywords:** PCOS, IL-39, inflammation, cytokines, biomarker, multivariable logistic regression, ROC analysis

## Abstract

**Background/Objectives**: Polycystic ovary syndrome (PCOS) is a common endocrine disorder associated with chronic low-grade inflammation. Interleukin-39 (IL-39), a newly identified cytokine, has been implicated in immune regulation; however, its role in PCOS remains unclear. This study aimed to evaluate serum IL-39 levels in patients with PCOS and its potential as an adjunctive inflammatory biomarker candidate. **Methods**: This case–control study included 44 patients with PCOS diagnosed according to the Rotterdam criteria and 44 age-matched in the control group. Serum IL-39 levels were measured using enzyme-linked immunosorbent assay (ELISA). Clinical and laboratory parameters were recorded. Group comparisons were performed using appropriate parametric and non-parametric tests. Correlation analysis was conducted using Spearman’s coefficient. Multivariable logistic regression analysis was performed to identify independent predictors of PCOS. Receiver operating characteristic (ROC) analysis was used to assess discriminative performance. **Results**: Serum IL-39 levels were significantly higher in the PCOS group compared to control group (*p* < 0.001). No significant correlations were observed between IL-39 and other clinical or laboratory parameters. In multivariable analysis, IL-39 was independently associated with PCOS. ROC analysis showed that IL-39 had moderate discriminative ability (AUC = 0.74), with 68% sensitivity and 70% specificity. The combined model including IL-39, body mass index (BMI), luteinizing hormone (LH), and age demonstrated improved performance (AUC = 0.78), with higher sensitivity (86%) and negative predictive value (81%). **Conclusions**: IL-39 levels are elevated in PCOS and may represent a potential adjunctive inflammatory biomarker candidate. Its diagnostic performance improves when combined with other clinical parameters, supporting a multivariable approach in PCOS evaluation.

## 1. Introduction

Polycystic ovary syndrome (PCOS) is a metabolic and endocrine disease that occurs with a frequency of 3–10% among women of reproductive age [1]. PCOS is characterized by menstrual irregularities, oligo/anovulation, and hyperandrogenism and is associated with infertility, hirsutism, diabetes mellitus, dyslipidemia, cardiovascular diseases, and insulin resistance [2]. It is also suggested that the presence of chronic low-grade inflammation in these patients may be the cause of atherosclerotic processes [3]. Current studies suggest that PCOS is not only a hormonal disorder but may also be associated with chronic low-grade inflammation [4].

Recent evidence increasingly highlights the role of immune dysregulation and chronic low-grade inflammation in the pathogenesis of PCOS. Alterations in circulating cytokines, immune cell activation, and inflammatory mediators have been demonstrated in affected women, suggesting that immune-inflammatory pathways contribute to metabolic and endocrine abnormalities observed in PCOS [5,6]. Evidence in the literature suggests a potential relationship between PCOS and inflammatory cytokines. For example, women with PCOS have been shown to exhibit higher concentrations of several inflammatory mediators, such as C-reactive protein or interleukin-18 [7]. Some proteins, cytokines and chemokines are dysregulated in patients with PCOS patients. Omentin-1 is an adipokine with insulin resistance and anti-inflammatory effects. Omentin-1 levels have been shown to be decreased in patients with PCOS [8]. Interleukin-39 (IL-39) is a cytokine belonging to the interleukin-12 (IL-12) family, known to be associated with inflammatory and immune-mediated processes [9]. The IL-12 family consists of five subunits. IL-39 includes interleukin-12 alpha subunit p19 (IL-23p19) and Epstein–Barr virus-induced gene 3 (3EBI3), which activate signal transducer and activator of transcription 1 and 3 (STAT1 and STAT3) [10]. This activation plays a role in the inflammatory response. At the same time, pro-inflammatory cytokines stimulate IL-23p19 expression in endothelial cells [11]. This increased expression suggests a potential role for IL-39 in inflammation-related immune and vascular regulation. IL-39 has been found to be associated with some autoimmune and inflammatory diseases [12].

Recent studies have also emphasized that cytokines belonging to the IL-12 family play important roles in immune regulation, autoimmune diseases and their potential importance in inflammatory disorders [13,14]. Inflammation is considered as a key component of PCOS and studies in the literature regarding IL-39 suggest that this cytokine may play a role in inflammation. However, to the best of our knowledge, no previous study has specifically investigated the association between PCOS and circulating IL-39 levels.

We hypothesized that serum IL-39 levels are elevated in patients with PCOS and may be associated with inflammatory pathways involved in PCOS and could represent a potential adjunctive biomarker candidate. Therefore, the aim of this study was to compare serum IL-39 levels in patients with PCOS to those in women without PCOS.

## 2. Materials and Methods

This prospective observational study was conducted in the gynecology outpatient clinic of Recep Tayyip Erdogan University Faculty of Medicine Training and Research Hospital between 1 November 2024 and 30 November 2025. Women diagnosed with PCOS were included in the study group and women with regular menstrual cycles included in control group. The PCOS diagnosis was made using the 2003 Rotterdam criteria. These criteria include oligo- or anovulation, clinical and/or biochemical hyperandrogenism, and polycystic ovarian morphology on ultrasonography (≥12 follicles 2–9 mm or ovarian volume > 10 mL), and exclusion of other etiological factors. Patients with at least two of the first three criteria were diagnosed as PCOS. All blood samples were collected in the early follicular phase of the menstrual cycle (days 2–3) after overnight fasting to minimize hormonal variability. Complete blood count, thyroid-stimulating hormone (TSH), luteinizing hormone (LH), follicle-stimulating hormone (FSH), estrogen, and progesterone levels were measured. Transvaginal ultrasound was performed on the second or third day of the menstrual cycle. All ultrasonographic examinations were performed by the same experienced gynecologist using the same ultrasound system to reduce measurement variability. Exclusion criteria for patients in the study were women with malignancy, history of gestational diabetes, myoma uteri, endometriosis, ovarian cysts, postmenopausal, pregnancy, and congenital adrenal syndrome or infections. Women using hormonal contraception, metformin, insulin-sensitizing agents, systemic corticosteroids, or other hormonal medications were excluded from the study.

Laboratory Measurements of IL-39: After an overnight fast, 5 mL of peripheral blood was collected and centrifuged at 3000 rpm for 20 min. Serum samples were stored at −20 °C until analyses. All samples were collected in the morning (08:00–10:00). Serum IL-39 concentrations were measured using a commercially available human IL-39 ELISA kit (MyBioSource, San Diego, CA, USA) according to the manufacturer’s protocol. The sensitivity of the test is reported as 1.07 ng/L according to the manufacturer’s protocol. Absorbance was determined at 450 nm within 5 min using a microliter plate reader (Multiskan GO, Thermo Scientific, Waltham, MA, USA). IL-39 levels were measured with a standard curve generated by Titri ELISA Software (Version 5.06). The standard curve was then used to convert absorbance values to IL-39 concentration. All serum samples were analyzed in duplicate according to the manufacturer’s instructions, and the mean values were used for statistical analyses. Laboratory personnel performing ELISA measurements were blinded to clinical group allocation. Intra-assay and inter-assay coefficients of variation were below 10%, indicating acceptable assay reproducibility.

For this study, the sample size was calculated using the G*Power Software (Version 3.1.9.4). Based on a moderate effect size (d = 0.55), a statistical power of 80%, and a significance level of α = 0.05, the minimum required sample size was calculated as 72 participants (36 in the PCOS group and 36 in the control group) using an independent samples *t*-test. To account for potential dropouts, 8 additional participants were added to each group. Therefore, 44 individuals were included in each group, resulting in a total sample size of 88 participants.

Statistical Analysis: Statistical analyses were performed using Jamovi Software (Version 2.4.6). The normality of continuous variables was assessed using the Shapiro–Wilk test. Variables with normal distribution were expressed as mean ± standard deviation (SD), while non-normally distributed variables were presented as median (minimum–maximum). Comparisons between the PCOS and control groups were performed using the independent samples *t*-test for normally distributed variables and the Mann–Whitney U test for non-normally distributed variables. Effect sizes were calculated using Cohen’s d for parametric data and r for non-parametric data. Correlation analysis was conducted using Spearman’s rank correlation coefficient to evaluate relationships between IL-39 and clinical/laboratory parameters. Multivariable logistic regression analysis was performed to evaluate variables independently associated with PCOS. IL-39 values were log-transformed due to their skewed distribution. Considering the relatively limited sample size, regression findings were interpreted cautiously due to the potential risk of model overfitting. Multicollinearity was assessed using variance inflation factor (VIF) values. Results were expressed as odds ratios (OR) with 95% confidence intervals (CI). Receiver operating characteristic (ROC) curve analysis was performed to evaluate the discriminative ability of IL-39 and the multivariable model. The optimal cut-off values were determined using the Youden index. Sensitivity, specificity, positive predictive value (PPV), and negative predictive value (NPV) were calculated. A *p*-value of <0.05 was considered statistically significant.

Generative Artificial Intelligence (AI): ChatGPT (OpenAI, GPT-5 series) was used to assist with language editing, sentence restructuring, and improving overall clarity and readability of the manuscript. The AI tool was not used for data analysis, interpretation of results, generation of scientific content, or drawing conclusions. All scientific decisions, analyses, interpretations, and final responsibility for the content of the manuscript remain solely with the authors.

## 3. Results

**Figure 1:**
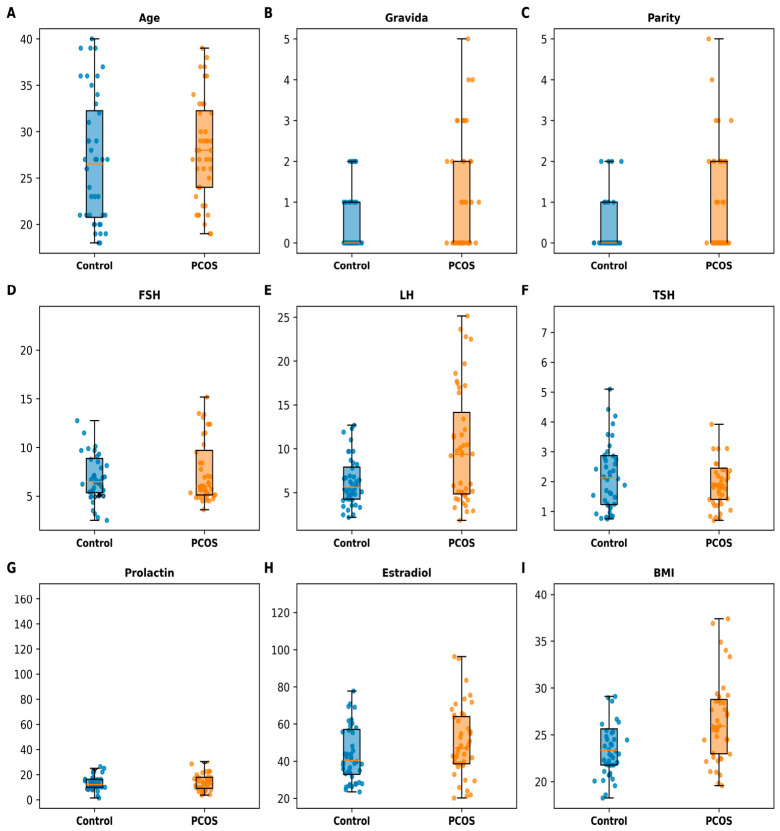
Distribution of demographic and hormonal variables in PCOS and control groups. Boxplots represent median and interquartile range, with individual data points overlaid. Significant differences were observed for LH (*p* = 0.023) and BMI (*p* = 0.005), while other variables showed no statistically significant differences (*p* > 0.05).

When comparing the PCOS and control groups, statistically significant differences were observed for luteinizing hormone (LH) (*p* = 0.023, 95% CI: 0.5–6.2) and body mass index (BMI) (*p* = 0.005, 95% CI: 0.9–4.8). In contrast, no statistically significant differences were observed for age, gravida, parity, FSH, TSH, prolactin, and estradiol levels (*p* > 0.05 for all) (Table 1 and Figure 1).

**Figure 2:**
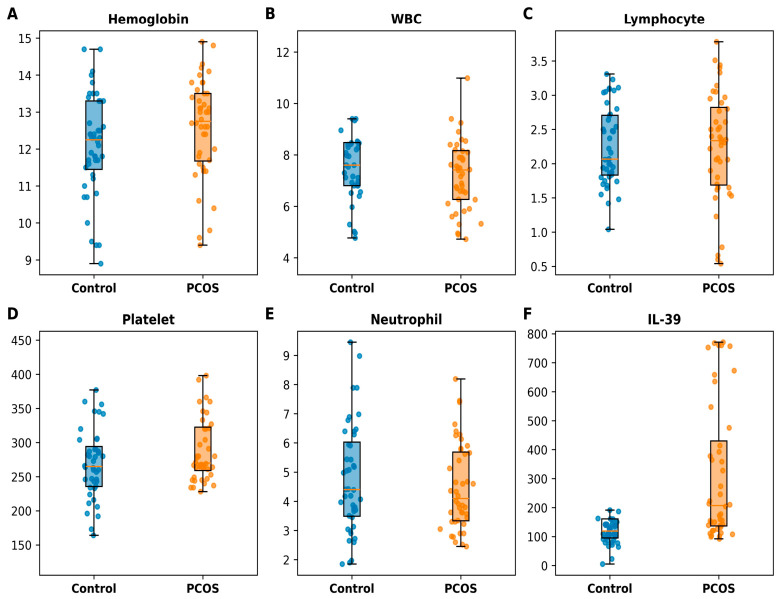
Distribution of hematological parameters and IL-39 levels in control and PCOS groups. Boxplots represent median and interquartile range with overlaid individual data points. A statistically significant difference was observed only for IL-39 (*p* < 0.001), whereas other variables showed no significant differences (*p* > 0.05).

When comparing hematological parameters and IL-39 levels between the PCOS and control groups, a statistically significant difference was observed only for IL-39. IL-39 levels were significantly higher in the PCOS group compared to the control group (*p* < 0.001, 95% CI: 21.49–233.10). In contrast, no statistically significant differences were found for hemoglobin, WBC, lymphocyte count, platelet count, or neutrophil levels (*p* > 0.05 for all) (Table 2 and Figure 2).

**Figure 3:**
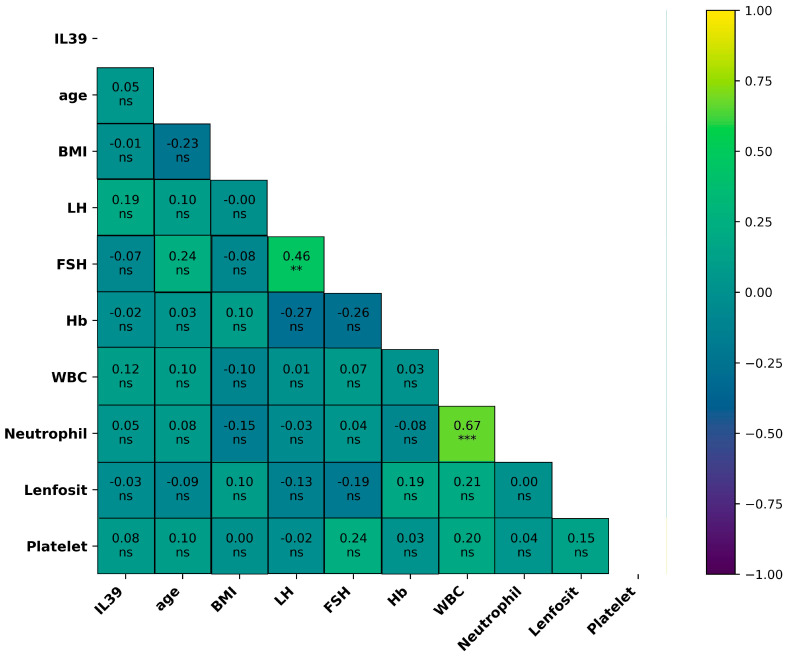
Spearman correlation matrix showing relationships between IL-39 and clinical/laboratory variables in the PCOS group. Only the lower triangle is displayed. Values represent correlation coefficients (r), with significance indicated as ns (*p* > 0.05), ** (*p* < 0.01), and *** (*p* < 0.001).

The correlation matrix demonstrated that serum IL-39 levels were not significantly associated with any of the evaluated demographic, hormonal, or hematological parameters in the PCOS group (all *p* > 0.05). In contrast, a statistically significant and moderate positive correlation was identified between LH and FSH levels (r = 0.46, *p* = 0.002), which is consistent with their physiological relationship. Additionally, a strong positive correlation was observed between leukocyte and neutrophil counts (r = 0.67, *p* < 0.001) (Table 3 and Figure 3).

Multivariable logistic regression analysis demonstrated that serum IL-39 levels were consistently and independently associated with PCOS across all models. In Model 1, IL-39 was significantly associated with increased odds of PCOS (OR = 3.03, 95% CI: 1.55–5.92, *p* = 0.001). This association remained stable after adjustment for BMI in Model 2 (OR = 3.12, 95% CI: 1.60–6.11, p < 0.001) and persisted even after further adjustment for LH and age in Model 3 (OR = 2.98, 95% CI: 1.53–5.78, *p* = 0.001) (Table 4).

**Figure 4:**
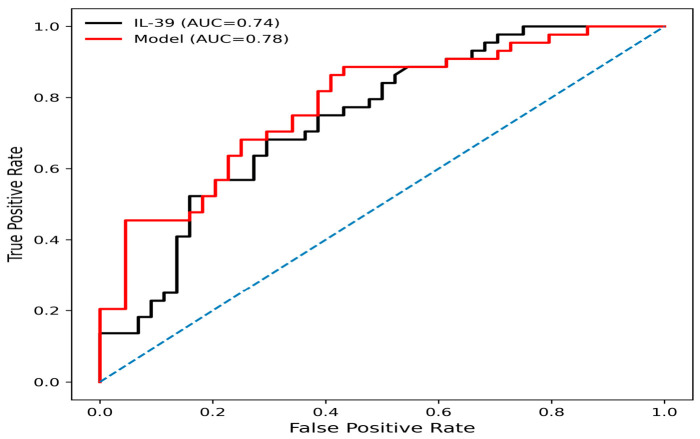
ROC curves for IL-39 (black) and the multivariable model (red). The combined model demonstrated improved discriminative performance compared to IL-39 alone.

ROC analysis demonstrated that IL-39 had moderate discriminative ability for distinguishing PCOS from controls (AUC = 0.74). At the optimal cut-off value of 149.3 ng/L, IL-39 showed a sensitivity of 68% and specificity of 70%, indicating moderate discriminative performance with limited standalone clinical utility. In contrast, the multivariable model incorporating IL-39, BMI, LH, and age exhibited improved discriminative performance (AUC = 0.78). This model achieved a higher sensitivity (86%) and NPV (81%), suggesting better ability to correctly identify PCOS cases and exclude non-PCOS individuals. However, specificity (59%) was relatively lower, indicating a moderate rate of false positives (Table 5 and Figure 4).

## 4. Discussion

The findings of the present study support the growing body of evidence suggesting that immune and inflammatory mechanisms play an important role in the pathophysiology of PCOS. While previous studies mainly focused on classical inflammatory cytokines such as IL-6, TNF-α, and IL-18, the current study expands this field by evaluating IL-39, a novel cytokine belonging to the IL-12 family. The data we obtained in our study show that circulating blood IL-39, a cytokine with pro-inflammatory properties, was found to be significantly higher in patients with PCOS compared with control group. This finding is consistent with other studies in the literature showing the presence of chronic low-level inflammation in PCOS patients [15].

Despite studies on PCOS, its pathophysiology is still not fully understood [16]. The pathophysiology of PCOS involves many complex interactions between genetic, metabolic, inflammatory, or endocrinological factors. Immune system activation and altered cytokine profile may contribute to disease development and progression. Studies have shown that immune cell activation is increased, cytokine balance is disrupted, and the immune response contributes to the pathophysiology of PCOS [17,18]. Although IL-39-specific mechanisms in PCOS have not yet been clearly elucidated, increasing evidence suggests that chronic low-grade inflammation and immune dysregulation contribute to the pathophysiology of PCOS. Since IL-39 belongs to the IL-12 cytokine family, which is involved in inflammatory and immune-mediated pathways, it may represent a potential immunological mediator associated with the inflammatory milieu observed in PCOS. However, current evidence regarding IL-39 remains limited and indirect, and its precise biological role in PCOS requires further mechanistic investigation.

IL-12 is a pro-inflammatory cytokine that plays a central role in regulating immune responses and promoting T-helper-1 (Th1) cell differentiation. Increasing evidence suggests that immune dysregulation and chronic low-grade inflammation are considered important components associated with the pathophysiology of PCOS; however, their precise causal role remains incompletely understood. IL-12 is primarily produced by antigen-presenting cells such as macrophages and dendritic cells, and it enhances inflammatory responses and cellular immunity by stimulating interferon-γ production [19]. Recent studies have shown that cytokines belonging to the IL-12 family may contribute to the altered cytokine profile observed in women with PCOS and may affect both metabolic and reproductive-related disorders through immune-mediated mechanisms [20]. In particular, activation of Th1-mediated immune responses and increased inflammatory signaling have been reported to be associated with insulin resistance and endocrine imbalance in PCOS [21]. Furthermore, the dysregulation of the IL-12 cytokine family has been demonstrated in various autoimmune and inflammatory diseases, supporting the hypothesis that immune-mediated mechanisms may play a role in the development and progression of PCOS [22]. Therefore, IL-12 and related cytokines may be important mediators linking immune activation with metabolic and reproductive function disorders in women with PCOS [23].

There are inflammation-related cytokines within the IL-12 family. A recent study indicates that IL-39 may be a pro-inflammatory cytokine associated with certain autoimmune or inflammatory diseases belonging to IL-12 family [24]. Although the biological functions of IL-39 have not yet been fully elucidated, experimental studies have shown that this cytokine can be secreted by B cells and may enhance neutrophil activation [25]. Elevated levels of interleukin-6 (IL-6), TNF-α, and IL-18 have been reported in patients with PCOS, and these mediators are thought to be linked with insulin resistance and obesity [26]. Insulin resistance is linked with an increased risk of developing type 2 diabetes mellitus and commonly observed in PCOS. Elevated circulating IL-39 levels have been observed in patients with type 2 diabetes mellitus [27]. Cytokines belonging to the IL-12 family are known to regulate interactions between innate and adaptive immunity, and their dysregulation has been reported in several metabolic and inflammatory diseases. Therefore, IL-39 may potentially be associated with immune activation and metabolic dysfunction observed in PCOS. In the present study, IL-39 levels were also found to be elevated in patients with PCOS, and may suggest a potential involvement in shared inflammatory and metabolic pathways.

IL-39 activates the STAT-1 and STAT-3 pathways, which are involved in pro-inflammatory response. STAT-3 activation, in particular, may be associated with both chronic inflammation and certain metabolic disorders [28]. From this perspective, increased IL-39 levels in PCOS patients may play a role in the immunological and inflammatory processes of the disease. Another possible explanation for the elevated IL-39 levels observed in this study may be related to immune-metabolic interactions, which are gaining increasing importance in the pathophysiology of PCOS. Today, PCOS is considered not only an endocrine disease but also a condition characterized by immune activation and chronic low-grade inflammation. It is known that cytokines belonging to the IL-12 family regulate communication between innate and acquired immune responses and may contribute to inflammatory signaling pathways involved in metabolic diseases. IL-39, composed of IL-23p19 and EBI3 subunits, has been shown to activate the STAT1 and STAT3 signaling pathways, which play a role in inflammatory and metabolic regulation. Activation of these signaling pathways may contribute to the metabolic disturbances observed in PCOS by promoting the recruitment of immune cells, cytokine production, and increased inflammatory responses. In this context, elevated IL-39 levels may reflect the activation of immune pathways associated with chronic inflammation and metabolic dysfunction in women with PCOS. suggests that to have and could provide new insights into the immunological mechanisms contributing to the development of PCOS.

In our study, BMI and LH levels were found to be significantly higher in the PCOS group. Obesity is a condition that may be associated with an inflammatory response and cytokine release. Obesity is strongly associated with the pathophysiology of PCOS and plays a significant role in exacerbating the inflammatory and metabolic disorders observed in affected women. It is now accepted that adipose tissue is not only an energy storage area but also a metabolically active endocrine organ capable of releasing numerous adipokine and inflammatory mediators that affect systemic metabolism and immune responses. In obese individuals with PCOS, the increased mass of adipose tissue leads to macrophage infiltration and increased release of pro-inflammatory cytokines such as IL-6 and tumor necrosis factor-α (TNF-α), contributing to a state of chronic low-grade inflammation. This inflammatory environment has been shown to disrupt insulin signaling pathways, thus contributing to the development of insulin resistance, one of the key metabolic disorders of PCOS. Insulin resistance can lead to increased androgen production in the ovaries and impaired follicular development. In addition, obesity-related adipokine imbalance can worsen the inflammatory response and metabolic dysfunction in women with PCOS through mechanisms such as decreased adiponectin levels and altered leptin signaling [29]. Consequently, obesity can exacerbate both the endocrine and metabolic features of PCOS through interrelated mechanisms such as chronic inflammation, insulin resistance, and adipokine imbalance [28]. However, in our study, the hemogram parameters were similar between the groups. In the correlation analysis, although IL-39 was elevated in the PCOS group, no correlation was found with clinical parameters such as BMI and LH. Furthermore, in multivariate logistic regression analysis, IL-39 was found to be associated with the presence of PCOS independently of BMI and LH levels. These findings suggest that elevated IL-39 levels may reflect a specific immune response independent of systemic hematological changes, obesity, or gonadotropin secretion disorders. Because BMI differed significantly between groups, residual confounding related to obesity-associated inflammation cannot be completely excluded despite multivariable adjustment. Although IL-39 levels were elevated in patients with PCOS, the absence of significant correlations with BMI and hormonal parameters suggests that its precise mechanistic contribution remains unclear.

According to the ROC analysis results, IL-39 has moderate discriminatory power in the diagnosis of PCOS (AUC: 0.74). It may not be sufficient on its own for clinical use. However, its discriminatory ability increases when evaluated together with BMI, age and LH. A multiparameter approach can be recommended. The improved sensitivity observed in the combined model suggests that IL-39 may have potential adjunctive value when evaluated together with clinical and hormonal parameters rather than as a standalone diagnostic marker. Nevertheless, it may be more appropriate to evaluate IL-39 in conjunction with other clinical, biochemical, and hormonal parameters rather than using it as a diagnostic marker alone.

We believe that our study may have some implications for clinical practice. Various interleukins have been investigated as therapeutic targets in some autoimmune diseases. Treatment studies with IL-39 antibodies in mice with lupus-like disease have yielded positive results [24]. Our study is the first to show that IL-39 levels are higher in PCOS patients than in the control group. IL-39 may represent a potential adjunctive inflammatory marker candidate in PCOS, although further validation is required. We believe that these data can contribute to the understanding of the pathophysiology of PCOS and can also pave the way for the investigation of whether it is a cytokine that can be used as a therapeutic target.

This study has some limitations. The single-center cross-sectional design and relatively limited sample size of this study limit the generalizability of the findings and preclude causal interpretation. Therefore, the present results should be considered preliminary and hypothesis-generating until confirmed by larger multicenter prospective studies. The absence of comprehensive metabolic and inflammatory parameters, including insulin resistance markers (such as HOMA-IR), CRP, waist circumference, androgen profiles, lipid measurements, and additional cytokines, limits the interpretation of IL-39 within the broader inflammatory and metabolic framework of PCOS. Although BMI was included in multivariable analyses, the significant difference in BMI between groups may still have contributed to residual confounding. At the same time, the fact that other cytokines that may reflect the inflammatory response were not examined simultaneously makes it difficult to determine the place of IL-39 in the inflammatory pathway. These limitations highlight the need for larger, multicenter, and prospective studies to further clarify the role of IL-39 in the pathophysiology of PCOS.

## 5. Conclusions

Circulating IL-39 levels are significantly higher in PCOS patients compared with control group. Further, larger-scale studies are needed to determine the potential clinical relevance of IL-39 in patients with PCOS. IL-39 may be a potential adjunctive inflammatory biomarker candidate and could contribute to the understanding of immune-related mechanisms in PCOS.

## Figures and Tables

**Table 1:** Comparison of demographic characteristics and baseline hormone profiles between the polycystic ovary syndrome (PCOS) and control groups. Data are presented as mean ± standard deviation (SD) for normally distributed variables and as median (minimum–maximum) for non-normally distributed variables, based on the Shapiro–Wilk normality test. Group comparisons were performed using the independent samples *t*-test for normally distributed variables and the Mann–Whitney U test for non-normally distributed variables. Effect sizes are reported as Cohen’s d for parametric variables and r for non-parametric variables. The 95% CI represent the difference between groups (mean difference for parametric variables and median difference for non-parametric variables). **PCOS**, polycystic ovary syndrome; **BMI**, body mass index; **FSH**, follicle-stimulating hormone; **LH**, luteinizing hormone; **TSH**, thyroid-stimulating hormone. A *p*-value of <0.05 was considered statistically significant. Bold footers used within tables indicate statistical significance.

Variable	PCOS (n = 44)	Control (n = 44)	*p*-Value	Effect Size	95% CI
Age	28.11 ± 5.55	26.50 ± 4.59	0.195	d = 0.08	[−1.2, 4.3]
Gravida	0 (0–5)	0 (0–5)	0.286	r = 0.06	[−0.5, 1.0]
Parity	0 (0–5)	0 (0–3)	0.367	r = 0.05	[−0.4, 0.9]
FSH (IU/L)	6.00 (3.60–21.60)	6.48 (2.51–23.50)	0.812	r = 0.03	[−1.8, 1.5]
LH (IU/L)	9.37 (1.83–25.14)	5.62 (2.17–19.70)	**0.023**	r = 0.33	[0.5, 6.2]
TSH (mIU/L)	1.90 (0.70–7.55)	2.13 (0.75–6.37)	0.815	r = 0.03	[−1.2, 0.9]
Prolactin (ng/mL)	13.89 (3.67–81.79)	12.06 (1.40–164.00)	0.661	r = 0.09	[−3.5, 6.8]
Estradiol (pg/mL)	50.15 ± 18.69	40.38 ± 16.39	0.154	d = 0.13	[−2.1, 12.5]
BMI (kg/m^2^)	25.93 (19.57–40.06)	23.34 (18.26–35.34)	**0.005**	r = 0.28	[0.9, 4.8]

**Table 2:** Comparison of complete blood count parameters and serum IL-39 levels in PCOS and control groups. Data are presented as mean ± standard deviation for variables considered normally distributed and as median (minimum–maximum) for non-normally distributed variables. Variables were considered normally distributed only when both groups satisfied normality assumptions. Effect sizes are reported as Cohen’s d for parametric variables and r for non-parametric variables. CI represent mean or median differences between groups. A *p*-value < 0.05 was considered statistically significant. Bold footers used within tables indicate statistical significance.

Variable	PCOS (n = 44)	Control (n = 44)	*p*-Value	Effect Size	95% CI
Hemoglobin (g/dL)	12.75 (8.90–14.90)	12.10 (9.60–14.80)	0.168	r = 0.18	[0.05–1.25]
WBC (×10^9^/L)	7.42 (4.72–11.50)	7.60 (4.10–12.90)	0.236	r = −0.13	[−1.27–0.51]
Lymphocyte (×10^9^/L)	2.26 ± 0.80	2.25 ± 0.57	0.95	d = 0.01	[−0.28–0.30]
Platelet (×10^9^/L)	270.00 (228.00–447.00)	268.66 (154.00–420.00)	0.087	r = 0.18	[−15.50–33.51]
Neutrophil (×10^9^/L)	4.10 (2.45–8.19)	4.77 (1.85–9.45)	0.494	r = −0.07	[−1.39–0.61]
IL-39 (ng/L)	207.00 (92.02–770.85)	118.80 (5.27–729.03)	**<0.001**	r = 0.41	[21.49–233.10]

**Table 3:** Correlation analysis between serum IL-39 levels and clinical and laboratory parameters in the PCOS group. Values are presented as Spearman correlation coefficients (r) with corresponding *p*-values in parentheses. Bold footers used within tables indicate statistical significance.

Variable	IL-39	Age	BMI	LH	FSH	Hb	WBC	Neutrophil	Lymphocyte	Platelet
IL-39	**1**	0.05 (0.73)	−0.01 (0.96)	0.19 (0.21)	−0.07 (0.65)	−0.02 (0.91)	0.12 (0.45)	0.05 (0.77)	−0.03 (0.86)	0.08 (0.61)
Age	0.05 (0.73)	**1**	−0.23 (0.13)	0.10 (0.52)	0.24 (0.11)	0.03 (0.83)	0.10 (0.52)	0.08 (0.61)	−0.09 (0.56)	0.10 (0.52)
BMI	−0.01 (0.96)	−0.23 (0.13)	**1**	−0.00 (0.99)	−0.08 (0.60)	0.10 (0.52)	−0.10 (0.50)	−0.15 (0.33)	0.10 (0.52)	0.00 (0.98)
LH	0.19 (0.21)	0.10 (0.52)	−0.00 (0.99)	**1**	0.46 **(0.002)**	−0.27 (0.08)	0.01 (0.94)	−0.03 (0.85)	−0.13 (0.39)	−0.02 (0.89)
FSH	−0.07 (0.65)	0.24 (0.11)	−0.08 (0.60)	0.46 **(0.002)**	**1**	0.03 (0.83)	0.07 (0.66)	0.04 (0.80)	−0.19 (0.22)	0.24 (0.11)
Hb	−0.02 (0.91)	0.03 (0.83)	0.10 (0.52)	−0.27 (0.08)	0.03 (0.83)	**1**	0.03 (0.84)	−0.08 (0.61)	0.19 (0.22)	0.03 (0.84)
WBC	0.12 (0.45)	0.10 (0.52)	−0.10 (0.50)	0.01 (0.94)	0.07 (0.66)	0.03 (0.84)	**1**	0.67 **(<0.001)**	0.21 (0.17)	0.20 (0.19)
Neutrophil	0.05 (0.77)	0.08 (0.61)	−0.15 (0.33)	−0.03 (0.85)	0.04 (0.80)	−0.08 (0.61)	0.67 **(<0.001)**	**1**	0.00 (0.99)	0.04 (0.80)
Lymphocyte	−0.03 (0.86)	−0.09 (0.56)	0.10 (0.52)	−0.13 (0.39)	−0.19 (0.22)	0.19 (0.22)	0.21 (0.17)	0.00 (0.99)	**1**	0.15 (0.34)
Platelet	0.08 (0.61)	0.10 (0.52)	0.00 (0.98)	−0.02 (0.89)	0.24 (0.11)	0.03 (0.84)	0.20 (0.19)	0.04 (0.80)	0.15 (0.34)	**1**

**Table 4:** Multivariable logistic regression analysis of factors associated with PCOS. Model 1 included IL-39 alone; Model 2 included IL-39 and BMI; and Model 3 included IL-39, BMI, LH, and age. IL-39 values were log-transformed due to their skewed distribution. Odds ratios (OR) with 95% CI are presented. A *p*-value < 0.05 was considered statistically significant. Bold footers used within tables indicate statistical significance.

Variable	Model 1 OR (95% CI)	*p*-Value	Model 2 OR (95% CI)	*p*-Value	Model 3 OR (95% CI)	*p*-Value
IL-39 (log)	3.03 (1.55–5.92)	**0.001**	3.12 (1.60–6.11)	**<0.001**	2.98 (1.53–5.78)	**0.001**
BMI	—	—	1.17 (1.04–1.31)	**0.009**	1.16 (1.02–1.30)	**0.019**
LH	—	—	—	—	1.15 (1.03–1.29)	**0.013**
Age	—	—	—	—	1.01 (0.93–1.10)	0.789

**Table 5:** ROC analysis of IL-39 and the multivariable model for discrimination of PCOS. The optimal cut-off values were determined using the Youden index. Sensitivity, specificity, PPV, and NPV are presented for each model. The multivariable model included IL-39, BMI, LH, and age.

Model	AUC	Cut-Off	Sensitivity	Specificity	PPV	NPV
IL-39	0.74	149.3	0.68	0.70	0.70	0.69
Model 3 (IL-39 + BMI + LH + Age)	0.78	0.35	0.86	0.59	0.68	0.81

## Data Availability

The datasets used and/or analyzed during the current study are available from the corresponding author on reasonable request.

## Abbreviations

The following abbreviations are used in this manuscript:

PCOS	Polycystic ovary syndrome
IL	Interleukin
STAT	Signal transducer and activator of transcription
TSH	Thyroid-stimulating hormone
LH	Luteinizing hormone
FSH	Follicle-stimulating hormone
BMI	Body mass index
